# Differences in pulse rate variability with measurement site

**DOI:** 10.1186/s40101-020-0214-1

**Published:** 2020-02-21

**Authors:** Emi Yuda, Kento Yamamoto, Yutaka Yoshida, Junichiro Hayano

**Affiliations:** 1 Tohoku University Graduate School of Engineering, Aoba 6-6-05 Aramaki Aoba-ku, Sendai, 980-8759 Japan; 2 University of Tsukuba Graduate School of Comprehensive Human Sciences, 1-1-1 Tennodai, Tsukuba, Ibaraki, 305-8577 Japan; 3 Nagoya City University Graduate School of Design and Architecture, Kita Chikusa 2-1-10 Chikusa-ku, Nagoya, 464-0083 Japan; 4 Department of Medical Education, Nagoya City University Graduate School of Medical Sciences, 1 Kawasumi Mizuho-cho Mizuho-ku, Nagoya, 467-8601 Japan

**Keywords:** Pulse rate variability, Heart rate variability, Pulse wave, Electrocardiogram, Wearable sensor

## Abstract

**Background:**

Recently, attempts have been made to use the pulse rate variability (PRV) as a surrogate for heart rate variability (HRV). PRV, however, may be caused by the fluctuations of left ventricular pre-ejection period and pulse transit time besides HRV. We examined whether PRV differs not only from HRV but also depending on the measurement site.

**Results:**

In five healthy subjects, pulse waves were measured simultaneously on both wrists and both forearms together with single-lead electrocardiogram (ECG) in the supine and sitting positions. Although average pulse interval showed no significant difference from average R-R interval in either positions, PRV showed greater power for the low-frequency (LF) and high-frequency (HF) components and lower LF/HF than HRV. The deviations of PRV from HRV in the supine and sitting positions were 13.2% and 7.9% for LF power, 24.5% and 18.3% for HF power, and − 15.0% and − 30.2% for LF/HF, respectively. While the average pulse interval showed 0.8% and 0.5% inter-site variations among the four sites in the supine and sitting positions, respectively, the inter-site variations in PRV were 4.0% and 3.6% for LF power, 3.8% and 4.7% for HF power, and 18.0% and 17.5% for LF/HF, respectively.

**Conclusions:**

These suggest that PRV shows not only systemic differences from HRV but also considerable inter-site variations.

## Introduction

With the spread of wearable pulse wave sensors in recent years, pulse wave signals are used not only to measure pulse rate but also to analyze pulse rate variability (PRV) as a surrogate for heart rate variability (HRV) [1, 2]. For PRV under certain conditions, such as during sleep [3] and at rest [1, 2], the validity of PRV as a surrogate of HRV has been supported by some studies. However, there are many reports that the amplitude of the respiratory component of PRV obtained from the peripheral pulse wave is larger than that obtained from HRV of electrocardiogram (ECG), and the difference increases especially in the standing position [4, 5].

In contrast to HRV, which reflects variations in the ventricular myocardial electrical excitation cycle, PRV reflects variations in the intervals of the pressure waves generated by ventricular contractions and conducted through the arterial wall to measurement site. Therefore, PRV includes, in addition to HRV, the beat-to-beat variations in the pre-ejection period and pulse conduction time which are affected by respiration and autonomic neural activity.

However, these are two questions here. Question 1 is whether the effects of these mechanisms on PRV are simply to modify (amplify or attenuate) certain components existing in HRV or to generate variability not existing in HRV. Question 2 is whether these mechanisms result in only systemic differences between PRV and HRV, or also in inter-site variations among PRVs measured at different sites.

Of these, there is an interesting report for Question 1 from Constant et al. [6]. In 10 children with an implanted cardiac pacemaker, they recorded finger pulse wave during ventricular pacing at a fixed rate of 80 bpm and performed PRV spectral analysis. Despite that ECG R-R interval was constant and there was virtually no short-term HRV, a clear spectrum of respiratory fluctuation components was observed in PRV. Their studies show that respiration can “generate” the respiratory variability in PRV without HRV. Of Question 2, there were only a few studies. Nilsson et al. [7] recorded pulse wave signals at the forearm, finger, forehead, wrist, and shoulder to determine suitable locations for monitoring heartbeat and breathing. They found the best coherence with heartbeat at the finger and that with respiration at the forearm, but they failed to analyze the relationship between PRV and HRV or site-to-site difference among PRVs. Because pulse wave velocity decreases as arterial diameter decreases, slight difference in local vasculature can cause inter-site differences not only in pulse transit time but also in its variations. In the present study, we measured pulse waves at both wrists and both forearms simultaneously together with ECG and examined whether PRV differs not only from HRV but also depending on the measurement site.

## Results

Subjects were instructed not to move during the measurement to avoid any movement of the body affecting the sensing of the pulse wave and causing noise in the signal. Compliance with this instruction was confirmed with a built-in 3-axis acceleration sensor in the PPG sensor. At least during the time where the recorded data was used for analysis, the sensors did not detect any movement.

**Fig. 1:**
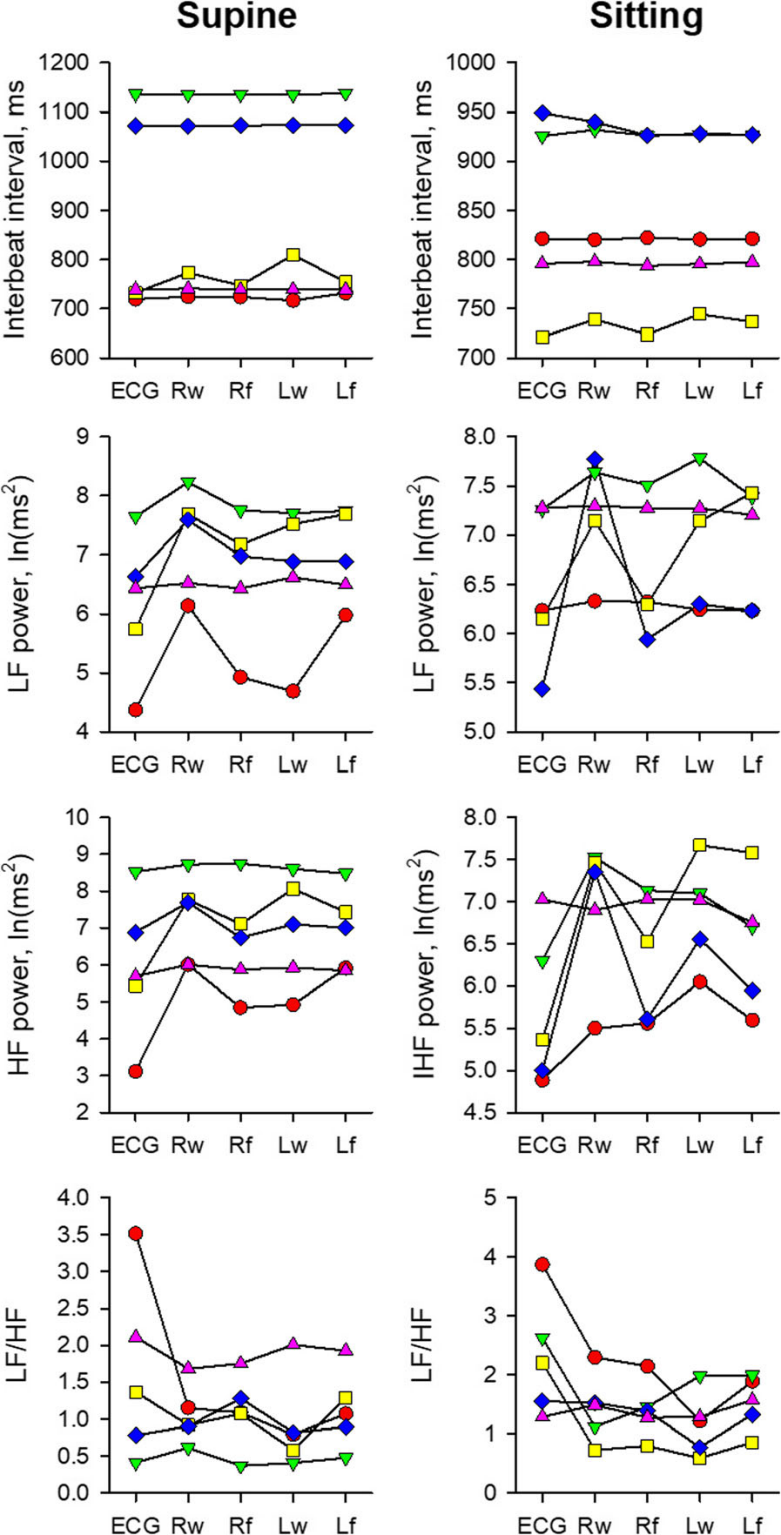
Mean interbeat intervals and variability measures of ECG R-R and pulse intervals at four different sites in five subjects. Different colors present different subjects and data of each individual subject are connected by lines for visibility. *HF* high-frequency component, *LF* low-frequency component, *Lf* left forearm, *Lw* left wrist, *Rf* right forearm, *Rw* right wrist

Figure 1 shows PRV measures obtained from pulse waves at the four different sites and HRV measures obtained from ECG in five healthy subjects. Although mean pulse interval and mean R-R interval did not differ significantly, the LF and HF power were greater and LF/HF were smaller in PRV than in HRV in both supine and sitting positions (Table 1).

**Table 1:** Comparison of pulse rate variability (PRV) and heart rate variability (HRV)

	Supine		Sitting		Signal (PRV vs HRV)		Signal × posture	
	PRV*	HRV	PRV*	HRV	*F*(1,42)	*P*	*F*(1,42)	*P*
Mean interval, ms	889 ± 64	880 ± 68	842 ± 64	843 ± 68	0.04	0.8	0.04	0.8
LF power, ln (ms^2^)	6.9 ± 0.3	6.2 ± 0.4	6.9 ± 0.3	6.5 ± 0.4	8.89	0.004	0.4	0.5
HF power, ln (ms^2^)	6.9 ± 0.4	5.9 ± 0.5	6.7 ± 0.4	5.7 ± 0.5	13.84	0.0006	0.01	0.9
LF/HF	1.1 ± 0.2	1.6 ± 0.3	1.4 ± 0.2	2.3 ± 0.3	13.97	0.0006	0.71	0.4

*HF* high-frequency component, *LF* low-frequency component

*Average over four sites

Data are least-square mean ± SE

Table 2 shows the coefficient of deviation (CD) of PRV measures from the corresponding measures of R-R interval and the coefficient of variance (CV) of PRV measures among four different sites. Mean pulse interval showed small CDs (1.2 ± 2.6% and 0.1 ± 1.4% in the supine and sitting positions, respectively) and small CVs (0.8 ± 1.1% and 0.5 ± 0.3%). In contrast, the power of PRV frequency components showed large deviations from those of HRV (13.2 ± 14.4% and 7.9 ± 10.8% for LF power and 24.5 ± 30.6% and 18.3 ± 15.3% for HF power on average over four sites). The CDs of the HF power were greater than those of the LF power (*P* = 0.002 and < 0.0001). LF/HF of PRV showed even larger negative deviations from that of HRV (− 15.0 ± 38.7% and − 33.0 ± 29.6%). Similarly, mean pulse interval showed a small inter-site difference (CV, 0.8 ± 1.1% and 0.5 ± 0.3% in the supine and sitting positions). In contrast, the LF and HF power showed large CVs, and the CVs of LF/HF were even lager.

**Table 2:** Deviation from HRV measures and site-to-site variations of PRV measures

	Mean interval	LF power	HF power	LF/HF
Supine				
CD, %				
Left forearm	1.0 ± 1.3	15.3 ± 18.3	26.1 ± 38.8	− 10.7 ± 34.7
Left wrist	2.1 ± 4.7	9.2 ± 12.4	22.9 ± 28	− 27.3 ± 37.5
Right forearm	0.6 ± 0.9	8.9 ± 10.3	18 ± 24.7	− 10.8 ± 47.7
Right wrist	1.3 ± 2.4	19.6 ± 16.9	31.1 ± 38.0	− 11.3 ± 44.4
Average of 4 sites	1.2 ± 2.6	13.2 ± 14.4	24.5 ± 30.6	− 15.0 ± 38.7
CV, %	0.8 ± 1.1	4.0 ± 3.5	3.8 ± 3.2	18.0 ± 6.3
Sitting				
CD, %				
Left forearm	0.0 ± 1.6	7.2 ± 9.9	15.5 ± 16.9	− 25.7 ± 32.7
Left wrist	0.2 ± 1.9	7.8 ± 8.0	22.1 ± 16.6	− 43.3 ± 30.8
Right forearm	− 0.4 ± 1.1	3.3 ± 3.6	12.2 ± 7.7	− 32.8 ± 26.2
Right wrist	0.5 ± 1.3	13.2 ± 17.8	23.3 ± 19.8	− 30.2 ± 35.6
Average of 4 sites	0.1 ± 1.4	7.9 ± 10.8	18.3 ± 15.3	− 33.0 ± 29.6
CV, %	0.5 ± 0.3	3.6 ± 3.8	4.7 ± 3.0	17.5 ± 5.2

*CD* coefficient of deviation, *CV* coefficient of variance, other abbreviations are explained in foot note to Table 1

Data are mean ± SD

Table 3 shows the significances of the effects of laterality (left and right), position (forearm and wrist), posture (supine and sitting), and their interactions on CDs and the effects of posture on CVs of PRV. Although the CD of mean pulse interval was greater and CD of LF/HF was smaller in the supine position than in the sitting position, the CDs of the other PRV measures showed no significant difference with laterality, position, or posture. The CV of any PRV measures showed no significant difference with posture.

**Table 3:** Effects of laterality, position, and posture on CD and CV of PRV measures

		Mean interval		LF power		HF power		LF/HF	
	DF	*F*	*P*	*F*	*P*	*F*	*P*	*F*	*P*
CD									
Laterality (left and right)	1, 31	0.58	0.4	0.16	0.6	0.01	0.9	0.49	0.4
Position (forearm and wrist)	1, 31	2.34	0.1	1.30	0.2	1.40	0.2	1.05	0.3
Posture (supine and sitting)	1, 31	6.22	0.01	2.61	0.1	1.14	0.2	5.29	0.02
Laterality × position	1, 31	0.05	0.8	3.92	0.05	0.78	0.3	1.34	0.2
Laterality × posture	1, 31	0.31	0.5	0.03	0.8	0.01	0.90	0.10	0.7
Position × posture	1, 31	0.15	0.7	0.21	0.6	0.11	0.7	0.00	0.9
Laterality × position × posture	1, 28	0.29	0.5	0.33	0.5	0.25	0.6	0.02	0.8
CV									
Posture	1, 4	0.75	0.4	0.03	0.8	0.2	0.6	0.05	0.8

*DF* degree of freedom. The other abbreviations are explained in foot note to Tables 1 and 2

## Discussions

To answer the question whether the measures of PRV show only systemic differences from those of HRV or they also show differences among measurement sites, we measured pulse wave at four different sites simultaneously and compared PRV and HRV measures. We observed that mean pulse interval showed only 1.2% and 0.1% deviations from mean R-R interval and 0.8% and 0.5% inter-site variations in the supine and sitting positions, respectively. In contrast, the power of PRV showed 13.2% and 7.9% deviations for the LF component and 24.5% and 18.3% deviations for the HF component from those of HRV in the respective positions. LF/HF showed even greater deviations of − 15.0% and − 33.0%. PRV measures also showed inter-site variations of 4.0% and 3.6% for the LF power, 3.8% and 4.7% for the HF power, and 18.0% and 17.5% for LF/HF in the supine and sitting positions. These observations suggest that there may be not only systemic differences between PRV and HRV measures, but also local differences in PRV measures depending on the pulse wave measurement site.

Theoretically, PRV is considered to be caused by fluctuations in cardiac autonomic nervous activity reflected in HRV, which are superimposed and modified by fluctuations in the pre-ejection period and pulse transit time due to mechanical and neurohumoral regulatory effects on the left ventricle and arterial system [1, 2, 5, 6]. PRV is observed even in patients with fixed rate ventricular pacing without HRV [6], and the difference between PRV and HRV is known to be amplified in the standing than in the supine position [1, 2, 5]. On the other hand, it has been unclear whether these factors generate only systemic differences between PRV and HRV or they also cause site-to-site differences in PRV and, if so, what is the magnitude of their contributions. Our observations indicated that systemic factors may cause a 10–25% difference in the LF and HF power and local factors cause a 4–5% variance among PRV measured at the forearms and wrists of the left and right arms.

The mechanism by which the difference between PRV and HRV occurs requires considerations not only of physiological factors but also of technical issues. The pulse wave signal is a smooth curve without distinct fiducial points like ECG R waves. Thus, there is a technical limit to measuring the pulse intervals with the same accuracy as the R-R intervals. To overcome this problem, we avoided to measure directly the pulse intervals, but instead used the method of PFDM [3] that extracts the instantaneous pulse interval as a continuous function. In a previous study using simulation data, we demonstrated that PFDM faithfully detects changes in pulse interval even when it changes greatly and is resistant to changes in pulse height and baseline fluctuations of signals [3]. In addition, the extracted pulse interval function exhibits an almost flat frequency characteristic in which the transfer gain in the range of 0 to 0.43 Hz falls in the range of 0.97 to 1.02. Furthermore, since PFDM does not depend on the detection of the peaks, the pulse interval estimation accuracy does not depend on the sampling frequency; the accuracy for pulse wave signal with 20-Hz sampling frequency is equivalent to that of R-R interval from ECG sampled at 125 Hz. PFDM does not provide pulse intervals of individual beats but directly estimates continuous pulse interval function. This may be a disadvantage of PFDM. In time series analysis such as a spectrum analysis, however, the beat-to-beat pulse interval time series is interpolated into a continuous function and resampled at equal time intervals. PFDM does not need this step. Therefore, this can be said to be an advantage rather than a disadvantage of PFDM.

Another technical issue of PRV is body motion artifact. Body movement may cause noise due to poor adhesion of the sensor to the skin or penetration of external light and may also cause artifacts in the pulse wave signal due to inertial movement of the peripheral blood volume. There are several studies on technologies against the motion artifacts in pulse wave. Fukushima et al. [8] have improved the accuracy of pulse rate estimation with an algorithm that uses three-axis acceleration data to eliminate motion artifacts in reflective photoplethysmographic (PPG) sensors. Kagawa et al. [9] has developed an array-type sensor that increases the level of pulse wave detection, detects noise based on the average value of the pulse wave spectrum amplitude, and excludes data at that time. Maeda et al. [10, 11] studied the optimal mounting pressure for reflective PPG sensors to reduce body motion artifacts. They showed that among the upper arm, the forearm, and the wrist, the measurement of the upper arm having the smallest acceleration due to the arm swing is least affected by body motion artifact. In these studies, the optimal pulse wave measurement site was examined from the viewpoint of removing motion artifacts, but the effect of the measurement site on PRV was not examined. In this study, the pulse wave was measured at rest and no movement of the PPG sensors was detected by the built-in acceleration sensors. Thus, the effect of body motion artifacts was considered to be small. Nevertheless, we observed that the PRV obtained from the pulse wave still showed variation between measurement sites.

From the considerations above, the cause of the inter-site difference in PRV observed in the present study is unlikely to be due only to the technical problems of the pulse interval measurement, but is more likely to be due to local physiological property. It is not simply, however, explained by the distance from the heart to the measurement site, because the inter-site difference in CD was not explained by laterality (left or right) or position (forearm or wrist) of measurement. The possible mechanisms may include the anatomic differences in local vascular architecture and the functional variations caused by local autonomous vasomotions [12].

In this study, the number of subjects and the distribution of age and gender were limited, and the measurement site of pulse wave was limited to the wrist and forearm. Therefore, the deviation of PRV from HRV and the variation between measurement sites observed in this study do not estimate population values. To estimate the quantitative differences between PRV and HRV, and the proportion of contribution of systemic and local factors, we need to include pulse wave measurements at fingers, earlobes, and face. Additionally, although we recorded pulse wave signals at four different sites and ECG simultaneously, the exact timing of the signals could not be adjusted in millisecond order because each signal was recorded on a separate device. Determining the exact mechanism that causes inter-site differences in PRV requires accurate spatiotemporal analysis of pulse wave signals with perfectly synchronized recordings at different sites.

## Conclusions

We studied PRV obtained from pulse wave signals measured at four different sites and compared them with HRV obtained from ECG. We observed that PRV differed from HRV and also differed between sites. Our findings suggest that PRV is not only affected by systemic factors that cause differences from HRV but also influenced by local factors that cause site-to-site differences. These observations also suggest the importance of researches on PRV-specific biological information not available from HRV, rather than the substitutability of PRV as a surrogate for HRV.

## Methods

### Subjects

We studied five healthy subjects (age, 30 ± 7 years, two females), who gave their written informed consent to participate this study. The protocol of this study was approved by the Research Ethics Committee of Nagoya City University Graduate School of Medical Sciences and Nagoya City University Hospital (No. 60-18-0093).

### Measurements

Pulse waves were recorded with a wearable bracelet-type reflective PPG sensor (APM02, Suzuken, Nagoya, Japan). The pulse wave was measured from the reflection intensity of the green light exposed to the skin. In the sensor module, the pulse wave was digitized at 200 Hz and bandpass filtered from 0.6 to 4.0 Hz. The gain of the initial- and post-stage amplifiers was 1000x and 2000x. The signal-to-noise ratio was 1000. The output frequency of the final pulse wave signal was 32 Hz. The PPG sensor also incorporated a 3-axis accelerometer.

ECG was recorded with a bioelectric amplifier (Biotop Mini, East Medic Co., Ltd., Kanazawa, Japan) at 500 Hz with a bandpass filter between 10 and 1000 Hz.

### Protocol

Experiments were performed between 11:00 and 13:00 in a quiet room air-conditioned at 24 ± 3 °C. The pulse wave sensors were attached on the dorsal side of the wrist and forearm of both arms, and ECG electrodes with CM5 leads were attached on the chest wall. Data were recorded in the supine and sitting positions for 10 min each. During the sitting position, subjects relaxed their arms in pronation and placed on the desk. They were also instructed not to move during the measurement to avoid any movement of the body affecting the sensing of the pulse wave and causing noise in the signal.

### Data analysis

Pulse interval was measured by the method of pulse frequency demodulation (PFDM) [3]. PFDM is a time series analysis method developed for assessing instantaneous pulse frequency continuously from pulse wave signal, which has no distinct fiducial point for measuring beat intervals. In this method, the pulse interval is not directly measured, but the pulse wave signal is regarded as a cosine function and its amplitude and instantaneous frequency (pulse rate) are determined by the complex demodulation method [13, 14]. The accuracy of instantaneous pulse frequency measurement by PFDM including the robustness to the fluctuations of baseline and pulse height has been reported elsewhere [3].

In the present study, 10-min continuous pulse frequency data obtained from the four measurement sites in each body position were converted into continuous pulse interval data and resampled equidistant time interval so that 1024-point pulse interval time series data were obtained.

From 10-min ECG data, beat-to-beat R-R interval data were obtained by a fast peak detection algorithm that determined temporal position of all QRS complexes [15]. The results of QRS complex detection were reviewed with ECG wave forms displayed on a computer screen with the marker of detected R wave positions. All errors of QRS detections were edited and non-sinus beats (QRS complexes without the preceding normal P wave in the range of 0.12–0.20 s) were marked as it. R-R interval data were interpolated only using those comprised continuous sinus rhythm beats, interpolated with a step function, and resampled so that 1024-point R-R interval time series data were obtained.

After calculating mean intervals for 10 min, Hanning window was applied on the pulse and R-R interval data and fast Fourier transformation was performed. From the power spectrum of each data, the power of low-frequency (LF, 0.04–0.15 Hz) and high-frequency (HF, 0.15–0.45 Hz) components and LF-to-HF ratio (LF/HF) were computed.

### Statistical analysis

The differences in measures between pulse interval and R-R interval were evaluated by ANOVA with SAS Mixed procedure (SAS Institute, Cary, NC, USA) with source signal (pulse interval or R-R interval) and posture (supine or sitting) as fixed effects and subject as random effect.

To evaluate the effects of laterality (left or right), position (forearm or wrist), and posture (supine or sitting) on the magnitude of deviation of pulse interval measures from the corresponding measures of R-R interval, we introduced the CD defined with the following equation:

$$ CD\left(\%\right)=100\times \frac{x-y}{y} $$

where *x* represents a measure of pulse intervals and *y* represents the corresponding measure of R-R intervals. To evaluate the effects of posture (supine or sitting) on the variations in the measures of pulse intervals among four sites, we introduced the CV defined with the following equation:

$$ CV\left(\%\right)=100\times \frac{SD}{E} $$

where *E* and *SD* represent average and standard deviation of measures at four sites.

The effects of laterality, position, posture, and their interactions on CD and the effect of posture on CV were evaluated by ANOVA with SAS Mixed procedure. In these analyses, the power of frequency components was converted into natural logarithmic values. *P* < 0.05 was used as the criterion of statistical significance.

## Data Availability

The datasets used for the current study are available from the corresponding author on reasonable request.
